# A silent public health threat: emergence of Mayaro virus and co-infection with Dengue in Peru

**DOI:** 10.1186/s13104-021-05444-8

**Published:** 2021-01-21

**Authors:** Miguel Angel Aguilar-Luis, Juana del Valle-Mendoza, Isabel Sandoval, Wilmer Silva-Caso, Fernando Mazulis, Hugo Carrillo-Ng, Yordi Tarazona-Castro, Johanna Martins-Luna, Ronald Aquino-Ortega, Isaac Peña-Tuesta, Angela Cornejo-Tapia, Luis J. del Valle

**Affiliations:** 1 School of Medicine, Research and Innovation Center of the Faculty of Health Sciences, Universidad Peruana de Ciencias Aplicadas, Lima, Peru; 2 Laboratorio de Biologia Molecular, Instituto de Investigación Nutricional, Lima, Peru; 3 Subregión Morropon Huancabamba, Dirección Regional de Salud de Piura (DIRESA), Piura, Peru; 4 Escuela Profesional de Genética Y Biotecnología. Facultad de Ciencias Biológicas, Universidad Nacional Mayor de San Marcos, Lima, Peru; 5 Barcelona Research Center for Multiscale Science and Engineering, Departament D’Enginyeria Química, EEBE, Universitat Politècnica de Catalunya (UPC), Barcelona, Spain

**Keywords:** Arbovirus, Alphavirus, Mayaro virus, Dengue, PCR, Peru

## Abstract

**Objective:**

To describe frequency and clinical characteristics of MAYV infection in Piura, as well as the association of this pathogen with DENV.

**Results:**

A total of 86/496 (17.3%) cases of MAYV were detected, of which 54 were MAYV mono-infection and 32 were co-infection with DENV, accounting for 10.9% and 6.4%, respectively. When evaluating monoinfection by MAYV the main groups were 18–39 and 40–59 years old, with 25.9% and 20.4% respectively. Co-infections were more common in the age group 18–39 and those > 60 years old, with 34.4% and 21.9%, respectively. The most frequent clinical presentation were headaches (94.4%, 51/54) followed by arthralgias (77.8%, 42/54). During the 8-month study period the most cases were identified in the months of May (29.1%) and June (50.0%).

## Introduction

Mayaro virus (MAYV) infection is a neglected tropical disease that represents an important cause of acute febrile illness (AFI) in endemic areas. In 2019, PAHO declared an epidemiological alert due to the recent increase in outbreaks and the potential of this pathogen to become a significant public health issue [1]. The virus was first isolated from 5 patients in Trinidad and Tobago during the outbreak in the county of Mayaro in 1954 [2]. Outbreaks throughout the South American amazon basin have been reported [3], as well as imported cases in Europe [4].

MAYV is a positive-sense single-stranded RNA virus of the Alphavirus genus [4]. This group is composed of seven viruses, which share common antigenic sites: Bebaru virus, Chikungunya virus, Getah virus, Semliki Forest virus, Ross River virus, O’nyong-nyong virus, and UNA virus. The antigenic similarities between these viruses have previously shown cross-reactivity in serological tests [5–7]; for this reason molecular assays are required for a precise diagnosis [8].

The first case of MAYV in Peru was reported in 1999 by Tesh et al. [9], and multiple outbreaks have been reported in the Peruvian territory throughout the years [10–12]. Peru has the 2nd highest number of cases reported in Latin America according to academic literature with 230 confirmed cases, following Brazil with 495 cases [13]. However, it is important to note that previous studies have shown that the real disease burden may be underestimated due to underreporting and misdiagnosis [14].

Alphavirus infections are mainly characterized by a broad range of non-specific signs and symptoms including fever, headache, rash, myalgias, among others [5, 14, 15]. However, Semliki group alphaviruses have been associated with arthralgias [16] and persistent incapacitating joint pain due to MAYV infection has been previously reported in a series of imported cases [17–19]. Accurate clinical differentiation between arbovirus and MAYV is a challenge for clinicians in endemic areas [20]; however, without sensitive and specific diagnostic tools, a precise etiological diagnosis cannot be easily achieved [14, 21].

A topic of great interest is the association between MAYV infection with other arboviruses, particularly Dengue virus (DENV). Sporadic cases of co-infection between MAYV and DENV have been previously reported [18, 22, 23]. However, several issues remain unanswered regarding the clinical presentation of co-infections between these pathogens. For example, it has not yet been characterized if co-infections exhibit a more specific clinical presentation or more severe symptoms. Furthermore, it has not been determined if co-infections suggest evidence of a shared vector.

Non-human primates play an important role as reservoirs for MAYV, maintaining the zoonotic cycle in the rainforest. [24]. Even though enzootic cycles are not fully characterized, canopy dwelling mosquitoes of the genus haemagogus are considered the main vectors [25–27]. Izurieta et al. [27] proposed that *Haemagogus* spp. was responsible for the sylvatic cycle of virus transmission and people living or working in the peri-urban area or forest peripheries, would provide the bridge to the urban setting.

The degree to which MAYV is adapted to infect more anthropophilic urban mosquitoes such as *A. aegypti*, *Ae. albopticus* and *Ae. scapularis* has only been evidenced in laboratories [28, 29]. However, single amino acid mutations in alphaviruses have been shown to improve adaption to mosquito species that are not normally considered the primary vector [30]. Consequently, MAYV urbanization poses an important risk to become a significant public health issue with the potential for epidemics in the same way CHIKV evolved over the years [31]. Our aim is described the frequency and clinical characteristics of MAYV infection in Piura, as well as the association of this pathogen with DENV.

## Main text

### Methods

#### Study location

A consecutive cross-sectional study was performed in six primary heath care centers between February and September of 2016 in the district of Morropon, Piura (Additional file 1: Figure S1). The department of Piura is located in the northern coast of Peru sharing boundaries with Ecuador to the north. It has a population of 1,856,809 (the second larger in Peru), with 79.3% in urban areas and 20.7% in rural areas, according to the last national census. This region is endemic for different pathogens responsible for AFI such as DENV, leptospirosis, among others.

#### Study subjects

Patients were recruited in the context of the syndromic surveillance program between February and September of 2016. All patients with a suspected AFI who attended the healthcare facilities within 7 days since the onset of quantified or not quantified patient-reported fever, were included. Patients with a temperature greater than 38 °C for < 7 days without an identifiable source of infection and associated with one or more of the following signs and symptoms were included: headache, myalgia, arthralgia, retro-ocular pain, lower back pain, rash, hyperoxia, odynophagia, nausea, emesis, abdominal pain, asthenia, syncope, hypothermia, jaundice, and others. The exclusion criteria were patients with an incomplete medical record and patients with an identifiable source of infection, such as acute upper respiratory tract infections, pneumonia, and urinary tract infections, among others.

#### Ethics statement

The study protocol was approved by the Research Ethics Board of the Hospital Regional Docente de Cajamarca, Peru. The samples were obtained in the context of the epidemiological/syndromic surveillance program according to the health directives of the National Center for Epidemiology, Disease Control and Prevention of the Ministry of Health of Peru. Therefore, it was exempt from informed consent.

#### Samples

A total of 496 samples were collected using Vacuette TUBE Serum Separator Clot Activator (Vacuette; Greiner Bio-One, Kremsmünster, Austria). All the samples were stored at − 80 °C after collection for molecular assays.

#### Real-time reverse transcriptase PCR amplification for the detection of MAYV and DENV

RNA extraction was performed using the High Pure RNA Isolation Kit (Roche Applied Science, Mannheim, Germany) following the manufacturer’s instructions; 200 μl of the serum samples was used. The viral RNA obtained was stored at − 80 °C until use.

Amplification by Real-time RT-PCR assay for the detection of MAYV was carried out using the primers and PCR conditions described by Aguilar-Luis et al. [32]. Amplification by Real-time RT-PCR assay for DENV was described by Leparc-Goffart et al. [33], and the PCR conditions were described by Alva-Urcia et al. [14].

#### Statistical analysis

Qualitative variables were reported as frequencies and percentages. Chi square test was performed to estimate statistical association between the variables, a value of p < 0.05 was considered significant. All analyses were processed with the IBM Statistical Package for the Social Sciences (SPSS) software version 21.0 (SPSS, Chicago, IL, USA).

Maps were created using QGIS 3.12.3 software. Data for creating the map were acquired from the Instituto Nacional de Estadística e Informática (https://www.inei.gob.pe/).

### Results

A total of 496 samples were collected during the study period. The detection of MAYV was performed with RT-PCR amplification of nsP1 for MAYV RNA. A total of 86/496 (17.3%) cases of MAYV were detected by this assay and among these: 54 were MAYV mono-infection and 32 were co-infection with DENV, accounting for 10.9% and 6.4%, respectively.

Table 1 summarizes the demographic characteristics of all the patients included in this study. The main age groups diagnosed with Mayaro virus were the patients between 18–39 and 40–59 years old, with 29.1% and 18.6% respectively. When evaluating monoinfection by MAYV, the main groups were 18–39 and 40–59 years old, with 25.9% and 20.4% respectively. Co-infections were more common in the age group 18–39 and those > 60 years old, with 34.4% and 21.9%, respectively. No significant differences were found when evaluating monoinfection and co-infections and the demographic variables studied.

**Table 1:** Demographics in patients with arboreal acute febrile illness by MAYV and DENV

Characteristics	Total cases n = 496 (%)	MAYV n = 86 (%)	Co-infections		p value*
			Only MAYV n = 54 (%)	DENV & MAYV n = 32 (%)	
Age					
< 5	39 (7.9)	8 (9.3)	5 (9.3)	3 (9.4)	0.7097
5–11	68 (13.7)	12 (14.0)	8 (14.8)	4 (12.5)	0.7646
12–17	56 (11.3)	10 (11.6)	8 (14.8)	2 (6.3)	0.2311
18–39	146 (29.4)	25 (29.1)	14 (25.9)	11 (34.4)	0.4042
40–59	109 (22.0)	16 (18.6)	11 (20.4)	5 (15.6)	0.5846
≥ 60	78 (15.7)	15 (17.4)	8 (14.8)	7 (21.9)	0.4043
Sex					
Male	226 (45.6)	43 (50.0)	30 (55.6)	13 (40.6)	0.1807
Female	270 (54.4)	43 (50.0)	24 (44.4)	19 (59.4)	0.1807

*Chi-square test was performed. p value < 0.05 was considered statistically significant

In regard to the clinical presentation, the most frequent symptom in MAYV infection were headaches (94.4%, 51/54) followed by arthralgias (77.8%, 42/54) (Table 2).

**Table 2:** Clinical symptoms in patients with MAYV and DENV infection confirmed by PCR

Clinical symptoms	Total cases n = 496 (%)	MAYV n = 86 (%)	Co-infections	
			Only MAYV n = 54 (%)	DENV & MAYV n = 32 (%)
Headache	404 (81.5)	77 (89.5)	51 (94.4)	26 (81.3)
Arthralgia	357 (72.0)	68 (79.1)	42 (77.8)	26 (81.3)
Myalgia	378 (76.2)	68 (79.1)	41 (75.9)	27 (84.4)
Retro-ocular pain	306 (61.7)	62 (72.1)	41 (75.9)	21 (65.6)
Hyporexia	305 (61.5)	56 (65.1)	36 (66.7)	20 (62.5)
Lumbar pain	246 (49.6)	45 (52.3)	29 (53.7)	16 (50)
Nausea/Emesis	226 (45.6)	41 (47.7)	25 (46.3)	16 (50)
Odinophagia	171 (34.5)	31 (36.0)	21 (38.9)	10 (31.3)
Rash	81 (16.3)	11 (12.8)	6 (11.1)	5 (15.6)
Thrombocytopenia	6 (1.2)	2 (2.3)	1 (1.9)	1 (3.1)
Chest pain/dyspnea	20 (4.0)	2 (2.3)	2 (3.7)	0 (0)
Epistaxis	8 (1.6)	2 (2.3)	2 (3.7)	0 (0)
Gingivorraghia	3 (0.6)	2 (2.3)	1 (1.9)	1 (3.1)
Hematocrit increase	5 (1.0)	2 (2.3)	1 (1.9)	1 (3.1)
Petechiae	11 (2.2)	2 (2.3)	0 (0)	2 (6.3)
Lipothymia	3 (0.6)	1 (1.2)	1 (1.9)	0 (0)
Sudden decrease in T ° or hypothermia	3 (0.6)	1 (1.2)	1 (1.9)	0 (0)
Cold Extremities / cyanosis	2 (0.4)	1 (1.2)	1 (1.9)	0 (0)
Hypotension	1 (0.2)	1 (1.2)	1 (1.9)	0 (0)
Conjunctival injection	3 (0.6)	1 (1.2)	0 (0)	1 (3.1)
Weak pulse	1 (0.2)	1 (1.2)	1 (1.9)	0 (0)
Cough	1 (0.2)	1 (1.2)	0 (0)	1 (3.1)
Persistent vomiting	5 (1.0)	1 (1.2)	1 (1.9)	0 (0)
BP differential < 20 MMHg	1 (0.2)	0 (0.0)	0 (0)	0 (0)
Echimoses	2 (0.4)	0 (0.0)	0 (0)	0 (0)
Shaking chills	3 (0.6)	0 (0.0)	0 (0)	0 (0)
Hemoptysis	1 (0.2)	0 (0.0)	0 (0)	0 (0)
Altered mental state	1 (0.2)	0 (0.0)	0 (0)	0 (0)
Vaginal bleeding	1 (0.2)	0 (0.0)	0 (0)	0 (0)
Hepatomegaly or jaundice	1 (0.2)	0 (0.0)	0 (0)	0 (0)
Dizziness	1 (0.2)	0 (0.0)	0 (0)	0 (0)
Melena	2 (0.4)	0 (0.0)	0 (0)	0 (0)

**Fig. 1:**
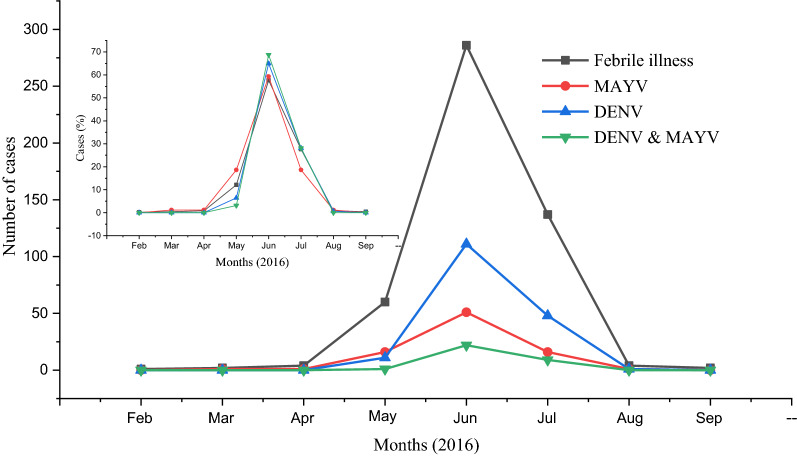
Monthly distribution of the numbers of total cases of acute febrile illness and the numbers of MAYV, DENV, and MAYV & DENV positive cases

Finally, an assessment of the monthly distribution of the MAYV cases detected during the 8-month period of study was performed (Fig. 1). During the 8-month study period the most cases were identified in the months of May (29.1%) and June (50.0%).

### Discussion

An important number of cases of MAYV were detected in patients with AFI that attended outpatient health-centers during this study. Approximately, a fifth of the total cases were diagnosed with MAYV, demonstrating that this pathogen may be circulating within the region and that peri-urban transmission may be ongoing. Moreover, approximately 6.4% of the total cases were co-infection between MAYV and DENV.

The association between MAYV and DENV is a topic of great interest for the scientific community, as both pathogens share common characteristics and can co-exist in a specific region. However, only two studies have previously reported co-infections of DENV and MAYV in the scientific literature, a child with an unspecified febrile illness, serologically diagnosed with MAYV, [23], and during an outbreak in Brazil [22]. Our study reports the first clinical characterization of patients co-infected with MAYV and DENV near the Peruvian Amazon basin for eight months. The frequency of unspecific symptoms among patients with mono-infection and co-infeccion were similar, including fever, myalgia, arthralgia, retroorbital pain, and headache. This finding further confirms the majority of symptoms amongst arboviral infections are non-specific, even when presenting as co-infections [9, 23, 34–36].

Pinheiro et al. [35] previously reported that the rash associated with MAYV infection appears on the fifth day of illness, suggesting its association with of humoral antibody appearance. In contrast, we found patients with a rash on the 3rd day of illness for MAYV, second day for co-infected patients, and the first day for DENV mono-infected patients.

Serologic evaluations for the detection of alphaviruses have shown cross-reactivity [8] as they belong to the Semliki complex serologic group [6, 7], denoting the importance of sensitive and specific molecular diagnostic methods such as RT-PCR. Previous studies report that viremia is detectable for up to 5 days post-infection [1]. However, we evidenced RT-PCR detection of MAYV up to 9 days in mono-infected patients and up to 7 days in co-infected patients. Our findings evidence that the window of detection for MAYV by RT-PCR could be longer than reported previously. Altogether, these characteristics make the RT-PCR an excellent diagnostic tool for the detection of MAYV during outbreaks.

Furthermore, our studied population showed no significant difference of positive cases between genders. A previous study determined that being male poses a risk for arboviral infections, given the higher occupational exposure [36]. Given that spillover zoonosis is considered the main source of recent arboviral outbreaks, this could have led to the peri-urban transmission of the disease [11]. Similarly, to our study, another outbreak in Brazil caused by peri-urban transmission of MAYV, showed that both genders were affected equally [22].

Additionally, we found a greater number of cases of both DENV and MAYV in May and July. This could be explained by some meteorological factors that could influence the vector expansion, behavior and biology. According to the meteorological national service (SENAMHI) the temperature and rainfall peak during the months of March and April; however, are still high during May and June, with a further decline in the later months. These factors altogether could enable a more easily transmission of the disease. Considering previous studies on the adaptability of alphaviruses to novel vectors [30], evidence of effective MAYV transmission by more urban anthropophilic mosquitoes in laboratory studies [28, 30, 33], and high aedic index reported in the studied region (aedic index 1–4%) [37], these findings may suggest that a common vector could be responsible for the transmission of both viruses during this outbreak. Further demographic and on-site vector studies are necessary to determine if the urbanization of MAYV is ongoing.

In conclusion, this study provides the first clinical characterization of patients co-infected with MAYV and DENV and also reports the first outbreak of MAYV-DENV co-infections, confirmed by molecular diagnostic methods. Our findings also provide further evidence that symptoms in co-infected patients are non-specific and that disease severity may not be associated with co-infections.

## Limitations

Some limitations of the study are that a majority of AFI cases could not be diagnosed with a precise etiology. Another limitation is that because of the design of the study, follow up could not be performed and long-term symptoms were not evaluated. Further, studies are required to better characterize the clinical picture and complications caused by co-infections between these two pathogens.

## Supplementary Information

**Additional file 1: Figure S1.** Geographic distribution of co-infections and mono-infections of MAYV and DENV in the Morropon districts, Piura-Peru.

## Data Availability

Abstraction format used in the study and dataset are available and accessible from the corresponding author upon request in the link: https://figshare.com/s/03fd58ac3f5cc806629f.

## Abbreviations

MAYV: Mayaro virus
DENV: Dengue virus
RT-PCR: Reverse transcription polymerase chain reaction
RNA: Ribonucleic acid
bp: Base pairs
